# A Technology-Based Classification of Firms: Can We Learn Something Looking Beyond Industry Classifications? †

**DOI:** 10.3390/e20110887

**Published:** 2018-11-18

**Authors:** Petros Gkotsis, Emanuele Pugliese, Antonio Vezzani

**Affiliations:** European Commission, Join Research Centre (JRC), 41092 Seville, Spain

**Keywords:** clustering, industrial classification, innovation studies, patents studies, industrial economics, scoreboard

## Abstract

In this work we use clustering techniques to identify groups of firms competing in similar technological markets. Our clustering properly highlights technological similarities grouping together firms normally classified in different industrial sectors. Technological development leads to a continuous changing structure of industries and firms. For this reason, we propose a data driven approach to classify firms together allowing for fast adaptation of the classification to the changing technological landscape. In this respect we differentiate from previous taxonomic exercises of industries and innovation which are based on more general common features. In our empirical application, we use patent data as a proxy for the firms’ capabilities of developing new solutions in different technological fields. On this basis, we extract what we define a Technologically Driven Classification (TDC). In order to validate the result of our exercise we use information theory to look at the amount of information explained by our clustering and the amount of information shared with an industrial classification. All-in-all, our approach provides a good grouping of firms on the basis of their technological capabilities and represents an attractive option to compare firms in the technological space and better characterise competition in technological markets.

## 1. Introduction

Firms are very heterogeneous organizations. Not only their characteristics—such as R&D investments [1,2,3] or firm size [4]—follow highly skewed distributions, they also operate in sectors with different competitive settings. An important issue for the empirical analysis of firms is therefore how to properly group them in order to allow for meaningful statistical comparisons. Indeed, firms compete on multiple layers and therefore their grouping cannot be deemed as independent from the research question at stake.

In this work, we follow an approach conceptually similar to Pavitt [5] to classify firms inductively according to the empirical regularities emerging from the data. Differently from previous contributions in line with Pavitt’s heritage [6,7,8], our classification exercise is based only on firms’ technological competences. The objective is to identify possible groups of direct competitors in the technology market, rather than grouping firms according to more general technological conditions characterising specific (groups of) sectors. In this sense, our aim is not to derive some common properties of knowledge within specific sectors [9] or “to describe the behaviour of innovating firms to predict their actions” [10] (p. 418), but to identify ‘technologically similar’ companies and group them together. Official sector classifications (e.g., NAICS, NACE, ISIC or ICB, the one used in this paper) classify firms on the basis of their economic activities. These are mainly related to their typical products, which may in turn be based on different technologies [11]; similarly a given technology may be used for different products. Therefore, traditional sector classifications may have a low explanatory power in defining the technological profiles of firms.

Of course, firms operating in the same sector share some macro-properties in their innovative behaviour; the taxonomy proposed by Pavitt in [5] was meant to capture both intra-sector similarities and inter-sector differences in the source, nature and impact of innovation. The aim was to contribute in filling a gap in the representation of technical change, by explicitly considering industrial differences in the technology creation and diffusion. The Pavitt taxonomy identifies four groups of sectors: *(i)* supplier dominated, traditional industries scarcely innovating and doing it mainly through machinery acquisition (process); *(ii)* Specialized suppliers, in a continuous interaction with their customers to provide innovations satisfying their needs; *(iii)* Science-based, looking for new discoveries within their R&D labs (e.g., pharmaceuticals, electronics or aerospace); *(iv)* Scale intensive, productivity driven firms with continuous interaction with their technology suppliers. Our analysis is based on the world top corporate R&D investors, which patent extensively in order to protect their intellectual property rights (IPRs) and fall mainly into categories 2 and 3. Therefore, in order to identify technological competitors at the global scale, we go a step beyond existing classifications and look through patent data at the firms’ actual technological competences to classify them into coherent groups.

In our opinion, a purely technological classification of firms based on patent data should be consistent with two stylized facts of the propensity to patent, the patent to R&D ratio: *(i)* the propensity to patent varies across industries [12], *(ii)* the propensity to patent varies substantially also within firms operating in the same industry [13]. The latter authors show, using the ISIC classification of a sample of top R&D investors, that the sample coefficient of variation of the patent propensity is about 4 and that for the Computer & electronic industry is even higher. A different technological focus of firms classified in the same sector may explain differences in the patent to R&D ratio and is also compatible with the persistent differences found in their R&D intensity, the R&D to sales ratio [14].

Differently from previous authors, we use a data driven approach to classify firms into groups with few ex-ante assumptions in order to reduce subjective interpretation. This is a very important feature considering that technological development causes continuous changes in the structure of industries. Our procedure allows for a fast adaptation of the classification to the changing technological landscape. In the past years, mathematical algorithms have been shown to be able to capture complex interactions between economic activities [15,16] and can help to explain the technological coherence of firms [17] or the innovation system at large [18,19]. This is usually done by moving from the evaluation of few dimensions, arbitrarily chosen by the researcher, to a high dimensional space looking in detail at the different activities performed by the unit object of the analysis. To this end, we will apply a clustering algorithm to the technological fields allotted to the patents filed by the firms of our sample. By looking at the technological fields in the patent portfolios of firms—where they are performing R&D and innovating—we derive what we define as a Technologically Driven Classification (TDC).

The main aim of this exercise is to “let the data speak” to reveal patterns not usually identified when using traditional classification schemes. As we said, we derive and discuss our classification on the top corporate R&D investors worldwide as reported in the Industrial Research and Innovation Scoreboard (http://iri.jrc.ec.europa.eu/scoreboard.html), a dataset produced by the JRC. These firms are the main actors in the global technological markets and therefore likely to represent the universe of firms competing there. Therefore, our approach does not aim at classifying out-of-sample companies based on their technological characteristics. However, in the appendix we will try to assess the out-of-sample performance of our algorithm; the tests reported are meant to check for the robustness of the technique used in this work.

Our sample is made of firms spread all around the world and operating in different sectors, but all are characterized by sizeable efforts in R&D and innovation. Moreover, the approach based on patent data is well suited for classifying highly innovative firms which resort to patenting for securing their IPRs. Therefore, it may not be the most appropriate to classify companies scarcely innovative or companies operating in sectors barely relying on patent protection (e.g., financial services).

### 1.1. Data

The data used in this work come from the 2015 edition of the EU Industrial R&D Investment Scoreboard (the Scoreboard), which is a dataset collecting information on the top 2000 corporate R&D investors worldwide. Altogether, these firms represent about 90% of world business R&D [3]. The Scoreboard is part of the European Commission’s monitoring activities of R&D investment trends by the private sector and of the factors that may affect innovative investments and performances. The firms in the Scoreboard are mainly big multinationals which are either independent companies or parents of a number of subsidiaries; these are defined as firms in which the parent company owns (directly or indirectly) at least 50% of shares.

Firms invest in R&D mainly to innovate and to increase their absorptive capacity by tapping into new knowledge and upskilling their human capital [20]. Firms, especially large ones, rely on IPRs to protect their innovations and guarantee economic returns from their R&D investment.

To analyse the technological outputs of the top R&D investors, we retrieve the patents they filed between 2012 and 2014 from PATSTAT: a relational database maintained and updated twice a year by the European Patent Office (EPO) which contains information about published patents from 83 patent authorities worldwide. Patents are very useful in innovation studies [21] because they contain a wealth of information about the different actors involved in the innovation process and are classified to different technological fields based on the International Patent Classification (IPC) scheme (http://www.wipo.int/classifications/ipc). The IPC is a hierarchical classification system containing, at the most detailed level, about 70,000 entries identified by classification symbols (IPC codes); these are allotted to patent documents. The IPC is backward compatible, is updated annually and it revised every three years to capture technological changes more effectively [22]. To identify the patent portfolio of firms, these should be linked with patent documents. To this end, the names of the top corporate R&D investors and of their subsidiaries were matched to the applicants’ names provided in published patent documents. We assume that the corporate structure of firms is relatively stable during the three years considered. The matching has been carried out on a country by-country basis using a series of algorithms contained in the Idener Multi Algorithm Linker (Imalinker) system developed by IDENER in 2013 (http://www.idener.es/). The matching exercise comprises a pre-processing procedure to ensure its consistency: firms’ names are harmonised and cleaned using country-specific ‘dictionaries’ based on country-level and language-related knowledge, this phase deals mainly with legal entity denomination and name variations. A series of string-matching algorithms, mainly token- and string-metric- based such as token frequency matching, and Levenshtein and Jaro-Winkler distances [23,24], were used to assess the matching accuracy. A final manual post-processing cleaning step assessing the proportion of non-matched firms and identifying new matches on a case-by-case basis was also performed (see [13]). The minimisation of false positives and the precision of the match were ensured through the selection of those pairs of company names/patent applicants that exceeded a predefined high-score threshold set in the algorithm. Patent portfolios were aggregated at the level of the headquarters; patents owned by a given subsidiary were thus fully attributed to the parent company of the group. Patent data are reported according to the earliest filing date and applicant, while the statistics rely on fractional counts to ensure that innovative output is not overestimated in the case of, e.g., shared ownership.

Depending on a number of factors and on market strategies that firms pursue, innovators may want to protect the very same invention in different countries. In this case, they need to file a set of related patent applications in each national or regional office where protection is sought. The first patent filing made to protect a given invention worldwide (the so-called “priority” filing) is often followed by (a series of) subsequent and related filings, thus giving birth to the so-called patent “family” [25]. To better reflect the inventive activities of top corporate R&D investors worldwide, the statistics presented here are based on families of patent applications filed at the five largest IP offices (IP5): the European Patent Office (EPO), the Japan Patent Office (JPO), the Korean Intellectual Property Office (KIPO), the State Intellectual Property Office of the People’s Republic of China (SIPO) and the United States Patent and Trademark Office (USPTO) (see [13] for further discussion of IP5 families). To be included in the sample, patent families should cover at least one IP5 office and another patent office in the world. This allows filtering out patents not likely to be valuable for firms, as they would not be protected in different jurisdictions. In addition, this choice protect us from the so called home bias [26] guaranteeing a balanced representation of the patent portfolios of firms from different countries.

Overall, 98% of top R&D-performing companies have been matched to at least one patent applicant, either directly or through one or more subsidiary firms, resulting in a sample of 1676 companies with over 117,000 IP5 patent families filed during the 2012–2014 period. Characterising and classifying companies based on the technology profile of their patent portfolio requires fractionally distributing patent families to the different technological fields they pertain to using the IPC classes as reported in the respective patent documents. In this work, the analysis is performed at the IPC 3-digit (IPC3) and at the IPC 4-digit (IPC4) level, which correspond respectively to 123 and 621 technology related dimensions (variables) for each firm entry. The dataset includes also firm level data like R&D expenditure, sales, and employment figures for the period under study, used to investigate further associations and tests. Each firm is classified into an industrial sector using the industry classification benchmark ICB classification scheme (http://www.ftserussell.com/financial-data/industry-classification-benchmark-icb). The firms in our sample belong to 38 different ICB 3-digit sectors (ICB3), which were used to make comparisons with the results of our proposed technology-based classification. The full sample of scoreboard companies and their subsidiaries is made of 600,000 firms and is representative of high tech companies active in developing cutting edge technologies worldwide.

### 1.2. The Clustering Exercise

In order to characterise and group firms on the basis of the similarities in the technological characteristics of their patent portfolios, we perform a cluster analysis using a hierarchical clustering algorithm. Hierarchical clustering is a type of clustering algorithm which does not require a priori knowledge of the number of clusters in which to divide the data into.

There exist two types of hierarchical clustering algorithms, namely top-down and bottom-up, which are also known as divisive and agglomerative hierarchical clustering algorithms. Divisive clustering starts by grouping the whole sample in one big cluster which is recursively split into two based on a specific method, until individual samples are reached. Agglomerative clustering instead starts by forming trivial singleton clusters which are successively merged at each step based on a specific criterion, until a big cluster containing the whole sample is reached. The results of the clustering can be visualised using a dendrogram, where horizontal lines at a specific level y correspond to a merger/split between samples having similarity y; in this way the whole history of successive mergers/splits can be reconstructed. The hierarchical clustering forms a hierarchical structure and can be more informative than other types of clustering. In addition hierarchical clustering is deterministic in the sense that the result of the grouping depends only on the distance metric criterion that has been used.

All this however comes at the cost of low efficiency and, as this type of algorithms can be computationally very intensive, a number of other clustering methods have been developed and proposed in the literature. One of the most commonly used alternative is the K-means clustering in which the objective is to minimize the average squared Euclidean distance of the cluster centres [27]. Each cluster centre is defined as the centroid of the observations forming the specific cluster. Ideally in this case the resulting clusters should be spherical and non-overlapping. In addition, the number of clusters K (the kardinality) is not known a priori and it has to be determined usually by performing a number of tests with different Ks in order to subsequently estimate the intra and inter cluster similarity of the resulting groupings. Another major difference between K-means and hierarchical clustering is that the result obtained from the former depends on the choice of the initial centroids which are randomly picked in order for the algorithm to start. Due to its simplicity and lower algorithmic complexity compared to hierarchical clustering algorithms, K-means is widely used for identifying groups within big samples where the use of hierarchical clustering is not a viable option. In this case the dimensions of the sample (2000 mother companies) were not prohibitive for the application of the hierarchical clustering. However, in Appendix A we also present the results of a clustering exercise based on K-means and we compare them with those presented in the main text.

Similarity between samples is assessed using a distance metric which in this work is the squared Euclidean distance between two firms *i* and *j* in the *n*-dimensional technology space:

(1) $$d_{i,j}=||\vec{i}-\vec{j}|{|}^{2}=\overset{n}{\underset{k=1}{\sum }}{\left(i_{k}-j_{k}\right)}^{2}$$

In Equation (1) *i_k_* and *j_k_* are the technology related coordinates of firms *i* and *j* in the *n*-dimensional technology space. Calculations have been performed using different data representations: (i) patent shares of firm *i* in technology *k*, where *i_k_* is a number between 0 and 1; (ii) binary representation, where *i_k_* = 1 in case company *i* owns patents in technology *k*; and, (iii) the relative technology advantage representation, where *i_k_* = 1 if firm *i* is *specialised* in technology *k*. In this last case we first define the relative technology advantage (RTA) of firm *i* in technology *k* as the share of patents of firm *i* in technology *k* over the average share of patents in technology *k* in the sample. An RTA > 1 means that firm *i* is specialised in technology *k*.

The clustering criterion used is the Ward’s method, which is a special case of the objective function approach [28] where two clusters are merged at each step based on the optimal value of an objective function: in this case the error sum of squares. The method is also known as the minimum variance method and is well suited for clustering multivariate continuous data. In this case, the objective function to minimise at each clustering step is ∆(A,B), the increase of the total sum of squares after two specific clusters *A* and *B* are merged:

(2) $$∆\left(A,B\right)=||\vec{i}-{\vec{m}}_{A\cup B}|{|}^{2}-||\vec{i}-{\vec{m}}_{A}|{|}^{2}-||\vec{i}-{\vec{m}}_{B}|{|}^{2}$$

In Equation (2) vectors m refer to the centre of cluster *A*, *B* and the resulting union cluster A∪B according to the respective index. The first term in the right hand side of Equation (2) corresponds to the square sum of errors of the resulting union cluster *A*, *B* while the other two terms correspond to the square sum of errors of the two initial clusters. Equation (2) can be written in the form:

(3) $$∆\left(A,B\right)=\frac{n_{A}n_{B}}{n_{A}+n_{B}}||{\vec{m}}_{A}-{\vec{m}}_{B}|{|}^{2}$$

where *n_A_*, *n_B_* correspond to the number of observations within clusters *A* and *B* respectively. This form of the objective function reveals another characteristic of the Ward method. Given that the increase in the total sum of squares depends both on the size of the two clusters *A* and *B*, and on the geometric separation of their centres, the method favours merging smaller pairs of clusters when the centres of these clusters are equally far apart as the centres of other pairs of larger clusters. The clustering algorithm used in this work is the linkage algorithm implemented in python scipy package. The clustering exercise was performed using patent data distributed over different technological fields as defined by the IPC4 level. Given our interest in investigating the links of our purely technology based classification scheme with the ICB3 one, we decided to use the results of the grouping at specific cut off levels: namely at five cluster families, 10, 20, 30 and finally 38 clusters. The latter is equal to the number of distinct groups in which our firms are classified according to the ICB3 classification. In what follows results are based on the binary representation of the data which perform better in the validation exercise, presented later on, compared to the results obtained using the other two representations (results are available upon request.).

In Appendix B we further assess the quality of the clustering results using an approach inspired by machine learning techniques, this way we evaluate the capacity of our clustering approach to predict the allocation of out-of-sample firms within the identified groups.

## 2. Results

### 2.1. Results of the Clustering

**Figure 1:**
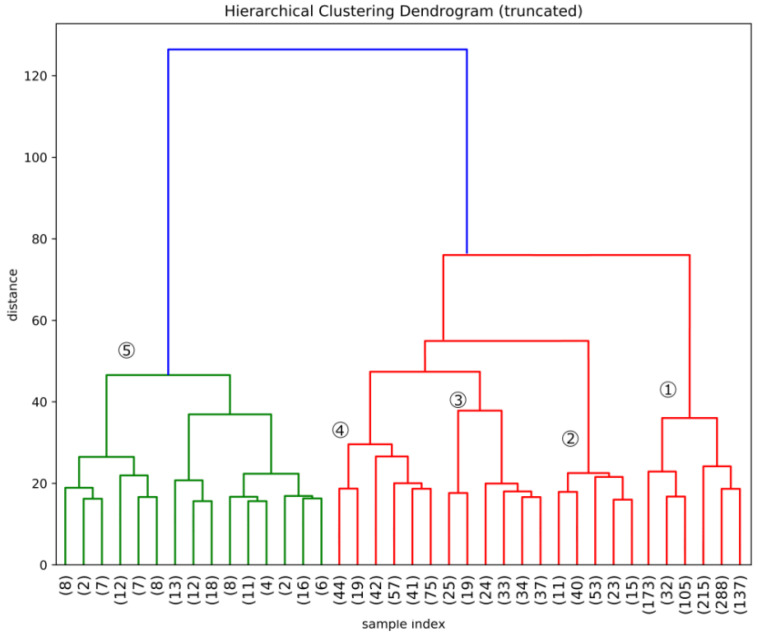
Representing the clustering up to 38. The 5 cluster families are highlighted in the dendogram.

The resulting hierarchical clustering for the case of the binary representation of the data matrix is represented in the dendrogram reported in Figure 1. There is not an obvious optimal point to stop the clustering, as any following cut increase the information.

As we said, for presentation purposes we stop the clustering exercise at 38 clusters which fall into 5 larger cluster families. We define this division of firms as the Technologically Driven Classification of firms.

### 2.2. Significance of the Clustering

First of all, we want to assess if our clusters share some common elements with the sector classifications, in particular with the Industrial Classification Benchmark (ICB) classification scheme. Since the ICB classification is not explicitly designed to capture technological development patterns between firms, we do not expect a near-perfect matching with our clusters, but we expect that, despite the presence of orthogonal information, the two are somehow related.

We measure the relatedness of the two classifications by following the procedure described in [29]. In particular, we compute the information present in the two classifications separately by measuring the entropy *H*(*X*) of each clustering *X*, and the common information between the two, *I*(*X*1, *X*2):

(4) $$H\left(X\right)=-\underset{x}{\sum }\frac{n_{x}}{n}log_{2}\frac{n_{x}}{n}$$

(5) $$I\left(X1,X2\right)=-\underset{x1}{\sum }\underset{x2}{\sum }\frac{n_{x1,x2}}{n}log_{2}\left(\frac{nn_{x1,x2}}{n_{x1}n_{x2}}\right)$$

where *n* is the total number of elements, *n_x_* the elements in cluster *x* and *n_x_*_1_, *n_x_*_2_ the elements that are in cluster *X*1 according to clustering *X*1 and in cluster *X*2 according to clustering *X*2.

The common information *I*(*X*1, *X*2) metric should be properly normalized with the information present in each cluster to give an idea of the distance between the two clustering methods. Two more refined clustering sets might share more information than two broader sets simply because they contain more information to begin with. Following [29], we define the Normalized Mutual Information (*NMI*) between clustering *X*1 and *X*2 as:

(6) $$NMI\left(X1,X2\right)=\frac{I\left(X1,X2\right)}{\sqrt{H\left(X1\right)H\left(X2\right)}}$$

**Figure 2:**
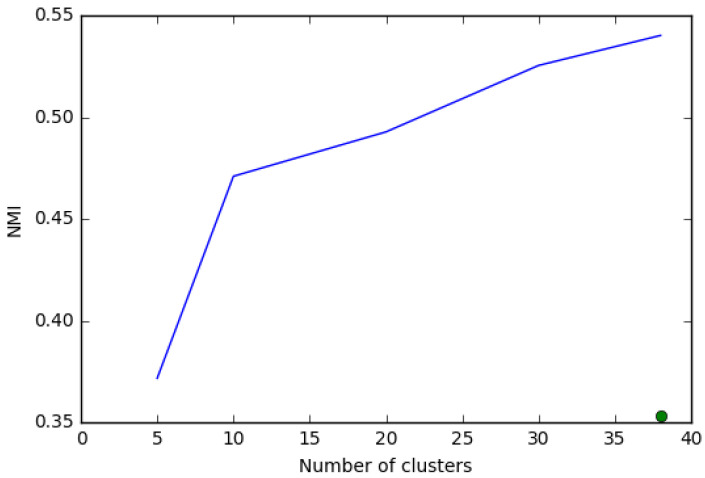
*NMI* with respect to the number of clusters. The blue line compares the clustering obtained using as input the IPC4 and the IPC3 codes. The green dot represents the *NMI* between the results of our clustering using the IPC4 codes and the ICB classification.

The mutual information between the TDC38 and the ICB3 is 36% of the total information (see the green dot in Figure 2). There is positive relationship, but the majority of information from our clustering is orthogonal to the one in ICB.

As a comparison, we see that a possible degree of freedom we have is the definition of the level of aggregation to be used in the IPC classification to characterize the input vector for the clustering algorithm. We repeat the clustering exercise using both classes (IPC 3-digits, 123 classes) and subclasses (IPC4-digits, 621 subclasses) and we compare the normalized mutual information present in the two clustering. In Figure 2 we present the results.

The mutual information between our clustering using the IPC3 and IPC4 is much higher than that with the ICB3. With the increase of the number of clusters, the clustering using different levels of detail on the information about technologies share an increasing proportion of information. All-in-all the clustering using the IPC3 and IPC4 provide quite similar information (54% at the 38 level), we opt for the more granular one, which performed better in the validity checks and allow us to better capture specific technological competences.

For a more direct comparison between our clusters and the ICB3 classification, we check if our clustering is more informative of innovation patterns, with respect to two external variables used for validation. In particular, we look with an ANOVA technique at the share of variance explained by the two clustering of: an innovation variable, patent propensity (defined as number of patents over R&D expenses), and a metric of economic performances, sales per employee (generally used to proxy labour productivity).

**Figure 3:**
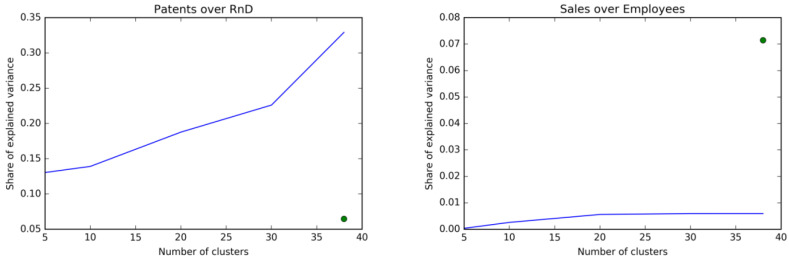
Share of variance of different variables explained by the clustering for different number of clusters (blue line) versus the share of variance explained by the ICB3 classification (green dot).

In Figure 3 we report in blue the share of variance explained by our clustering, for different numbers of clusters. The green dot represents the share of variance explained by the ICB3 classification. Our clustering works very well in explaining different innovation strategies of the firms: it properly groups firms together to capture 35% of the total variance of patent propensity against approximately 6% explained by the ICB3 classification. In other words, our clustering seems to work much better than the ICB3 classification in defining the technological similarities of firms.

On the other hand, the ICB3 classification performs better in capturing the variance of sales per employee (about 7%), which is an indicator capturing different firm dimensions than the technological one which is the object of our analysis. Consistently with its conceptualization, our classification outperforms the ICB3 in the technological domain while being unrelated to the economic performance dimension considered.

### 2.3. A Technological Classification of Firms

In this section we present some descriptive statistics to describe the salient characteristics of the clusters defined by the TDC and help the reader to understand the techno-economic significance of the clustering.

**Figure 4:**
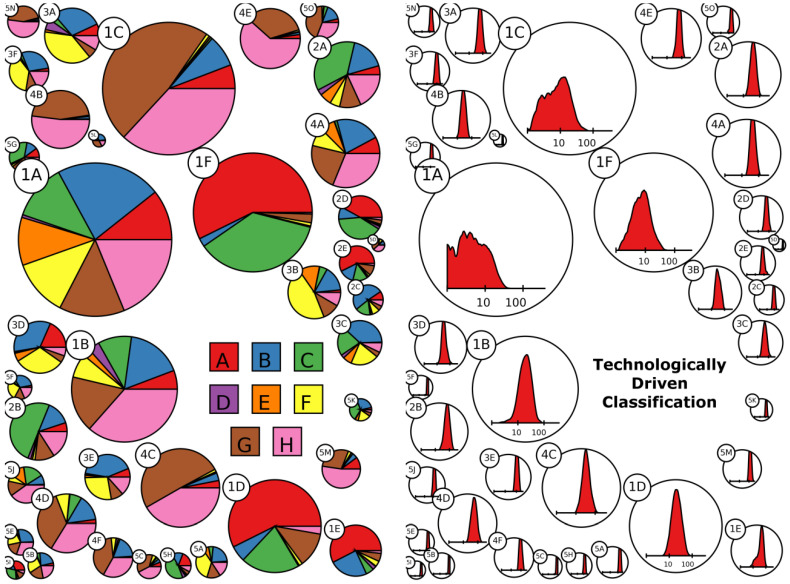
In the two subpanels we highlight the main characteristics of the 38 clusters. The size of the circles is proportional to the number of firms in the cluster. In the left panel, we show the share of patents in each of the 8 main sections of the IPC classification (IPC1): A—Human Necessities; B—Performing Operations, Transporting; C—Chemistry, Metallurgy; D—Textiles, Paper; E—Fixed Constructions; F—Mechanical Engineering, Lighting, Heating, Weapons, Blasting; G—Physics; H—Electricity. Sectors are divided in two groups related to the two main patenting industrial sectors: Health (sections A and C) and ICT (sections G and H). In the right panel, we show the distribution of firms’ technological diversification (# of IPC classes) in the clusters, presented in log-scale. Firms in clusters of Family 1 are not very diversified (around 10 fields), while the general diversification of our sample is generally very high. Firms in family 5 in particular are often characterized by a diversification above 100 different subclasses (out of 629 total subclasses in the IPC classification). From the right panel it is possible to see instead how the firms are divided in clusters with a similar breath of technological diversification (number of IPC4 codes in their patent portfolio). In particular, the clusters in family 1 show a low degree of diversification; in other words in these clusters are classified firms active in the development of a relative low number of technologies compared to the average Scoreboard firm.

In Figure 4, it is possible to see at a first glimpse how the clusters are characterized in terms of section specialization and diversification. For presentation purposes we represent technologies at the most aggregate level of the IPC classification: the IPC 1-digit (IPC1) level, which defines the eight main sections of the classification. Several aspects are immediately visible. First of all, the left panel shows that the clustering is able to identify and reclassify the two more numerous high tech sectors in the scoreboard sample: Information Technology/Electronics and Health, Chemical and Pharmaceutical. Indeed, several clusters have a large majority of G and H patents (Physics and Electricity—brown and pink) and C and A patents (Chemistry and Human Necessity—red and green). A third group of clusters is the one represented by F and B patents (Mechanical Engineering, Lighting, Heating, Weapons, and Blasting; and Performing Operations, Transporting—yellow and blue), related to the machinery and car industry, mostly located in cluster family 3.

We complement this information with the descriptive statistics reported in Table 1, where we also report the five cluster families to help understanding the major differences among the macro-categories our clusters belong to. Data, both across and within macro clusters are sorted by patent propensity.

Cluster family 5, reported on the top of the table, shows the highest average technological diversification; an average company in this macro-cluster has patents covering 182 different IPC4 codes. On average, companies in this macro-cluster are large in terms of R&D investments and have high patent propensity and R&D intensity. In the group there are both ICT-related and health-related clusters composed of highly research intensive firms. Each cluster composing the group identifies a well-defined technology sector, and the average size of the clusters is the smallest. For example, the cluster with the highest patent propensity (5D, a small circle on the centre of the right border in Figure 4) includes only two firms, Foxconn and Hon Hai Precision Industry Co. Ltd, both headquartered in Taiwan. These are part of the same group despite being listed separately on the Taiwanese stock market exchange; therefore are considered as two separate entities in the Scoreboard. While this may seem a bit odd result for our classification exercise, it nevertheless fits perfectly the technological specificity of this group. Indeed, these companies together make the largest electronic manufacturer in the world, working with major global players in the electronic and entertainment industries (between others, well known product includes: BlackBerry, iPad, iPhone, iPod, Kindle, Nintendo 3DS, Nokia devices, Xiaomi devices, PlayStation 3, PlayStation 4, Wii U, and Xbox One.). As to say that, these companies represent a unique technology business without real competitors. This specificity of Foxconn, often reported in journalistic economic analysis, has been highlighted by the clustering without any input from the researchers.

Cluster family 4 shows high average patent propensity coupled with relatively low technological diversification and R&D intensity. All the clusters in this family are focused on the development of ICT–related technologies. Families 3 and 2 are composed of firms of medium-high technology intensiveness and patent propensity. In particular, Family 3 is mainly composed by firms developing technologies related to transporting and advanced machinery, while Family 2 is composed of firms active in the development of chemical and material technologies.

Finally, the Family 1 cluster is composed of firms with low patent propensity. The TDC till gives some information on the firms in the clusters composing this family. In particular, we observe a division of the clusters specialized in ICT with respect to those in Health and Chemical. Finally, the algorithm groups in cluster 1A all the firms that are not of easy technological classification because of their low number of patents, these are mostly firms in the financial sector or other service companies (about 17% of the sample) not really relying on patents as a mean to protect their intellectual property despite sizable investments in R&D.

In general, our clusters nicely capture differences among firms beyond a broad technological diversification. In particular, the size of R&D investments, the R&D intensity, the most frequent technologies developed (technological specialization) and their shares over the total patent (technological concentration) varies between and within cluster families. For example, the cluster 5H identify a group of firms specialized in chemical technologies (C-codes) such as Bayer, with relatively high R&D investments and low patent concentration (18% of patents in the first 3 IPC4 codes). Differently, firms in cluster 3D (e.g., Boehringer Ingelheim)—in the low patent propensity, low diversification cluster family—appears to be smaller in terms of R&D investments, much more R&D intensive, more pharmaceutical oriented (class A, human necessities) and with a higher technological concentration (37% of patents in the first 3 IPC4 codes).

## 3. Conclusions

In this work we used clustering techniques to identify clusters of firms competing in similar technological markets. Starting from patent data, we are able to properly classify firms for which patenting is an important form of intellectual property protection. With this caveat in mind, we define clusters that properly capture the technological specificities of the top corporate R&D investors worldwide. Indeed, the clustering (re) groups together firms classified in different industrial sectors in a way that highlights their technological similarities.

While many of the characteristics found in this novel classification resonate with common elements found in innovation literature, in our exercise these properties emerged naturally and are not defined ex-ante by the researchers. Or methodology properly groups firms with similar technological profiles, thus allowing for a better characterisation of competition in technological markets. In other words, it allows adding a new layer of analysis in the studies of (innovative) firms’ performance. Moreover, the methodology provides a framework to identify emerging technological niches without a priori information of their characteristics. We believe therefore that our classification can be an important instrument for the analysis of the innovate elements of R&D intensive firms, both for academic purposes and to inform policy makers.

## Figures and Tables

**Table 1:** Descriptive statistics of the clusters.

Macro Cluster		Cluster	Patent Propensity	Diversification (# IPC4)	# of Firms	R&D	R&D Intensity	Top 3 Subclasses	% Top 3 IPC4
5	Patent Propensity: 0.91 # IPC4: 182 # of firms: 134 R&D: 1692 R&D intensity: 4.6%	5D	7.6	280	2	629	1.2%	G06F,H05K,H01R	36%
		5O	2.0	198	13	662	5.5%	G03G,H04N,G06F	30%
		5J	1.4	124	16	415	1.3%	H01L,E21B,H01M	44%
		5M	0.9	136	18	773	4.3%	H01L,G06F,A61B	39%
		5N	0.7	157	12	4140	9.6%	G06F,H04W,H04L	53%
		5C	0.7	289	7	3180	6.2%	H01L,G06F,H04N	33%
		5A	0.6	178	12	650	3.6%	F01D,E02F,F02C	15%
		5G	0.5	141	11	1715	10.3%	H01L,G02B,A61K	23%
		5K	0.5	136	6	366	0.5%	C22C,F25J,F17C	15%
		5F	0.4	285	7	3628	5.0%	H01M,B60W,B60R	14%
		5B	0.4	321	8	3209	4.0%	F01D,F02C,G06F	13%
		5E	0.3	205	8	3099	5.2%	H01L,H01M,B60W	12%
		5H	0.3	140	8	922	4.5%	C08G,C07C,C08L	18%
		5I	0.2	221	4	2582	5.7%	A61B,A61F,A61K	20%
		5L	0.03	134	2	937	2.5%	G06Q,G06F,B01D	24%
4	Patent Propensity: 0.54 # IPC4: 49 # of firms: 278 R&D: 273 R&D intensity: 5.0%	4F	1.2	93	19	273	6.3%	B41J,H01L,G06F	17%
		4D	0.8	57	42	124	4.6%	H01L,G02F,G06F	34%
		4E	0.5	66	44	606	7.5%	G06F,H04W,H04L	39%
		4C	0.4	36	75	331	4.1%	G06F,H04L,H04W	46%
		4A	0.4	47	57	127	2.3%	H01R,G01V,H01L	16%
		4B	0.3	27	41	165	13.9%	G06F,H01L,G11C	44%
3	Patent Propensity: 0.38 # IPC4: 62 # of firms: 172 R&D: 281 R&D intensity: 2.4%	3F	0.6	99	19	226	3.3%	F16C,H02K,B62D	21%
		3A	0.5	67	37	150	2.7%	D06F,A47L,B29C	9%
		3C	0.4	49	24	98	1.0%	C22C,F01D,C23C	19%
		3D	0.4	37	33	95	2.6%	A01D,F16D,B60T	21%
		3B	0.2	34	34	136	0.7%	E21B,F24H,F16K	20%
		3E	0.2	113	25	1132	4.3%	B60R,F16H,B62D	16%
2	Patent Propensity: 0.37 # IPC4: 60 # of firms: 142 R&D: 360 R&D intensity: 1.9%	2C	0.7	86	11	228	2.8%	B60C,A63B,C08L	41%
		2B	0.5	71	40	177	1.2%	H01L,C08L,C08G	18%
		2A	0.3	42	53	218	0.8%	H01M,B01J,C08F	17%
		2D	0.2	61	23	385	3.3%	A61K,C11D,A61Q	30%
		2E	0.2	78	15	1402	6.5%	A61M,A61K,A61F	36%
1	Patent Propensity: 0.11 # IPC4: 13 # of firms: 950 R&D: 146 R&D intensity: 3.0%	1E	0.4	42	32	154	1.3%	A61F,B65D,A47J	25%
		1B	0.2	21	137	73	1.5%	H01L,G02B,G06F	30%
		1D	0.1	23	105	441	10.7%	A61K,A61B,A61M	37%
		1C	0.1	10	215	155	3.9%	G06F,H04L,G06Q	46%
		1F	0.1	9	173	107	4.3%	A61K,C07D,A61P	56%
		1A	0.05	6	288	89	1.3%	H01L,B60N,H01R	14%

**Figure A1:**
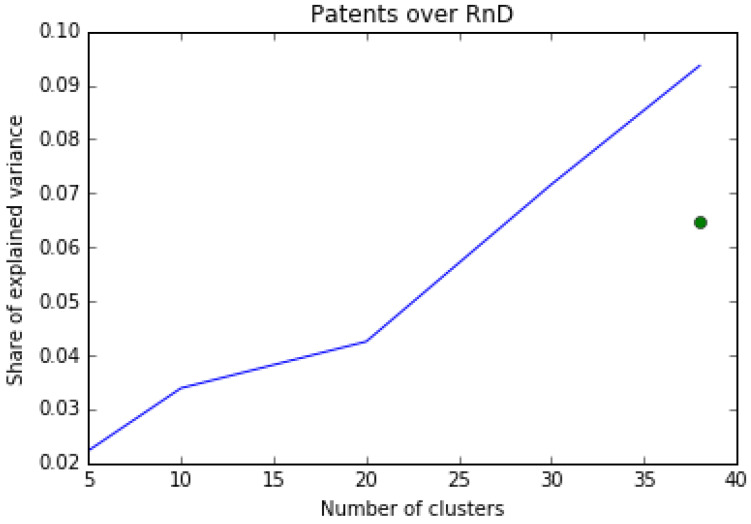
Share of explained variance of patent over R&D ratio explained by the K-means clustering with respect to the number of clusters (blue line), compared with the share of variance explained by the ICB3 classification (green dot).

**Figure A2:**
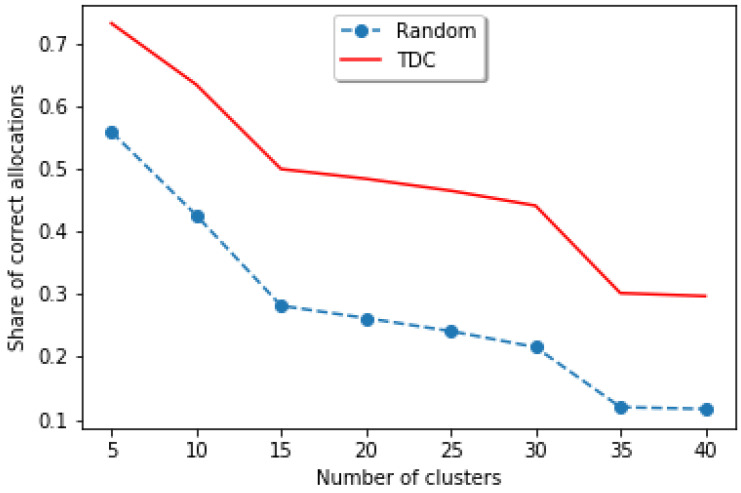
Share of successful predictions based on the nearest neighbour model (red solid line) and expected share of successful random predictions (blue dashed line).

## Appendix A. Results of K-Means Clustering

In addition to the grouping obtained from the agglomerative clustering using the Ward criterion, a grouping exercise based on K-means was also performed to compare with the results of the hierarchical clustering. To this end we run the K-means algorithm for K equal to 5, 10, 20, 30 and 38 and we check how informative of innovation patterns this clustering is compared to the ICB3 classification and the agglomerative clustering. In the following we look with an ANOVA technique at the share of variance of patent propensity, an innovation representative variable, explained by the different clustering approaches.

In Figure A1 the share of variance of the patent propensity variable explained by clusters obtained by K-means clustering is plotted as a function of the kardinality of the clustering. The green dot represents the share of variance explained by the ICB3 classification. The results shows that K-means clustering does not offer a big advantage in explaining the different innovation strategies of the firms compared to the ICB3 classification (9% versus 7%). This result should be compared to the results of the Ward’s method which properly groups firms together to capture 35% of patent propensity with 38 clusters (Figure 3). In other words our agglomerative clustering based on Ward’s criterion seems to outperform the K-means grouping in defining specific technological markets.

## Appendix B. Validation of the TDC Clustering Using the Nearest Neighbour

Grouping the Scoreboard companies on the basis of their technological capabilities is an interesting undertake in itself as it reveals patterns within the data characterising the technological capabilities of firms. However, the results of the agglomerative clustering are not informative about the potential to generalise our model and apply it to other companies outside of the Scoreboard sample. In order to validate the results of our grouping and assess the potential to generalise it to the technological characterisation and grouping of other firms, we use a nearest neighbour based approach.

First, we randomly split our initial sample of the 1676 firms into two equal subsamples of 838 firms. Then the agglomerative clustering used to identify the TDC is applied to the first subsample and the clustering results are used to estimate the coefficients of a multinomial regression. More specifically in the multinomial regression the group (cluster) ID obtained from the clustering exercise is the dependent regressor and the technological profile of the companies at the IPC4 level, are the independent variables. After estimating the coefficients using the firms in the first subsample (the training set) we use them to predict the cluster ID of the firms in the second sub sample (the validation set). The predicted group ID of each firm in the validation set is then compared to the group ID that it would have had, if it was assigned to the group ID of the closest firm in terms of technological characteristics from the training set. To this end we create a one to one correspondence between the firms in the two sets based on minimum Euclidean distance. Each firm in the validation set is thus associated with one and only one firm in the training set and “inherits” from it the group ID which is then used to judge the predictive power of the clustering.

Finally, we compare the share of successful predictions with the average number of successful predictions one would have expected in case of a random allocation of the 838 firms to the different clusters. We perform our analysis at different levels of aggregation by cutting the dendrogram obtained from the clustering exercise at heights identifying 5 to 40 clusters with steps of 5. The results of the validation are shown in Figure A2.

The estimated share of successful predictions based on the TDC scheme falls from approximately 75% when five clusters are considered down to 30% when the number of clusters is forty. At the same time randomly allocating firms at different clusters gives an average of 54% successful allocations at the level of 5 clusters which drops close to 10% at the level of 40 clusters. All-in-all, our approach shows discrete out-of-sample performances.
